# Predictors of metabolic syndrome among type II diabetic patients visiting public hospitals in Sidama Region, Ethiopia: unmatched case control study

**DOI:** 10.1186/s12872-026-05982-8

**Published:** 2026-05-16

**Authors:** Bereket Sisay Gebremichael, Amelo Bolka, Kaleb Philipos Rekiso, Assefa Philipos Kare

**Affiliations:** School of Public Health, Yirgalem Hospital Medical College, Yirgalem, Ethiopia

**Keywords:** Metabolic syndrome, Public hospitals, Type II diabetes mellitus, Sidama Region

## Abstract

**Introduction:**

Metabolic syndrome (MetS) is a series of health conditions, including insulin resistance, abdominal obesity, hypertension, and dyslipidemia. In diabetic patients, MetS elevates the risks of cardiovascular disease, stroke, and cardiovascular mortality. Its predictors are context-dependent, varying by diagnostic criteria and population characteristics, thus requiring localized studies to identify specific determinant factors.

**Objective:**

To assess predictors of MetS among type II diabetic patients (T2DM) visiting public hospitals in Sidama Region, Ethiopia, from January 25- March 25, 2025.

**Methods:**

An institutional unmatched case control study design was employed among 132 cases and 268 controls. Data were collected using a structured, interviewer-administered questionnaire adapted from the World Health Organization (WHO) STEPS instrument, complemented by laboratory investigations and standardized anthropometric measurements. MetS was diagnosed using the International Diabetes Federation (IDF) criteria. Bivariable and multivariable logistic regression models were fitted to determine predictors of MetS. Results were presented using adjusted odds ratios (AOR) with 95% confidence intervals (CI).

**Result:**

The mean age (± standard deviation) of the cases and controls was 56.9 (± 8.2) and 49.5 (± 8.1) years, respectively. MetS was found to be higher in female (61.4%) study participants than in male (38.6%). The identified predictors of MetS with 95% CI (AOR) were older age: 5.74 (2.56, 12.88), female sex: 2.91 (1.61, 5.26), urban residence: 2.59 (1.41, 4.75), monthly income > 3500 Ethiopian Birr: 4.30 (2.23, 8.28), family history of hypertension: 2.79 (1.47, 5.29), duration with DM: 5–9 years: 3.06 (1.57, 5.99) and ≥ 10 years: 3.61 (1.54, 8.48), and poor glycemic control: 3.93 (2.17, 7.13).

**Conclusion:**

The findings call for prioritizing targeted screening, lifestyle interventions, health education, and community-based programs to enhance the prevention, early detection, and management of MetS among T2DM patients.

## Introduction

Metabolic syndrome is a cluster of interrelated metabolic abnormalities that significantly increase the risk of cardiovascular diseases, T2DM, and all-cause mortality. These abnormalities include central obesity, dyslipidemia, hypertension, and insulin resistance [1]. Globally, the prevalence of MetS has risen dramatically over the past two decades, coinciding with increasing rates of obesity, physical inactivity, and urbanization [2]. It is a non-communicable condition, which presents a global public health and clinical challenge and the WHO estimates that more than one-quarter of the global adult population meets the diagnostic criteria for MetS [3].

T2DM is closely linked to MetS, primarily because insulin resistance serves as a key pathological mechanism underlying both conditions [4]. Patients with diabetes frequently display various components of MetS, which elevates their risk for cardiovascular complications when compared to those without diabetes [5]. This interplay between the two syndromes not only exacerbates glycemic control, making it more challenging to manage blood sugar levels effectively, but also complicates the overall management of the disease [6]. Consequently, this situation can result in increased rates of morbidity and mortality among diabetic patients [5].

The development of MetS among people living with T2DM is influenced by a complex interplay of genetic, metabolic, and lifestyle-related factors [4]. Excessive caloric intake, sedentary behavior, and obesity—particularly visceral adiposity—are major contributors [6]. In addition, other predictors such as age, gender, smoking, alcohol consumption, and psychosocial stress have been implicated in various studies [7]. Socioeconomic status and urban lifestyle transitions also play an important role in shaping metabolic health, particularly in low- and middle-income countries undergoing rapid urbanization [1].

Globally, 20–25% of adults have MetS, which doubles the risk of dying from cardiovascular disease and triples the risk of heart attack and stroke compared to those without the syndrome [8]. Low- and middle-income countries are witnessing an alarming rise in both T2DM and MetS due to lifestyle shifts, dietary changes, and reduced physical activity [9]. The dual burden of undernutrition and overnutrition, combined with limited access to healthcare, exacerbates the challenge. In sub-Saharan Africa, two-thirds of T2DM patients have MetS, with varying prevalence based on diagnostic criteria and population characteristics [10]. This heterogeneity highlights the importance of context-specific studies to understand local predictors and guide interventions [11].

Ethiopia, like many developing countries, is experiencing an epidemiological transition characterized by increasing noncommunicable diseases such as diabetes, hypertension, and cardiovascular disorders. Recent urbanization and lifestyle changes have led to a surge in obesity and sedentary behaviors among adults [12]. Despite the rising burden of MetS, T2DM and cardiovascular mortality and morbidity, data on the predictors of MetS among diabetic patients in Ethiopia remain limited [13]. Existing evidence suggests that MetS is common among Ethiopian T2DM patients, yet its underlying predictors vary across regions due to sociodemographic and environmental differences [14].

The Sidama Region, located in southern Ethiopia, has experienced rapid urban growth, dietary changes, and shifts in lifestyle patterns over the last decade. Public hospitals in Sidama are increasingly serving patients with diabetes and related metabolic complications [15]. However, there is limited research assessing the predictors of MetS in this population [16]. Recognizing region specific modifiable factors including lifestyle behaviors can guide both patients and healthcare providers in implementing effective management strategies. Moreover, exploring sociodemographic and behavioral correlates helps inform policy development for noncommunicable disease prevention and control at the community and institutional levels. Therefore, this study aims to assess the predictors of MetS among T2DM attending public hospitals in the Sidama Region.

## Methods and materials

### Study design, period and setting

An institution-based unmatched case-control study was conducted among Type T2DM patients attending the diabetic clinic for follow-up at two randomly selected public hospitals in the Sidama Regional State. As one of the twelve federal regions in Ethiopia, it lies just over 273 km from Addis Ababa. Home to approximately five million people, the region is structured into four zones, thirty rural districts, six town administrations, and a single city administration. The region’s healthcare system includes 553 health posts, 140 health centers, 17 primary hospitals, eight general hospitals, and one tertiary hospital, with DM treatment services accessible at all government hospitals. The investigation took place over a two-month period, commencing on January 25th and continuing until March 25th, 2025.

### Study population

#### Cases

Selected adult T2DM patients objectively diagnosed to have MetS at diabetic clinics in selected health facilities.

#### Controls

Selected adult T2DM patients in whom diagnosis of MetS is excluded using objective methods in selected public hospitals.

### Eligibility criteria

T2DM patients attending the diabetic clinics of selected hospitals on an outpatient basis during the study period and who volunteered to take part in the study constituted the study population. T2DM patients who were pregnant, abused excessive alcohol or other drugs, were on prolonged steroid anti-inflammatory medications or antipsychotic medications, or were diagnosed with hypothyroidism were excluded from the study. Adult patients confirmed to be HIV-positive and currently receiving antiretroviral therapy were also excluded.

### Sample size determination

The sample size for the study was determined using Epi Info 7 STAT CALC for an unmatched case-control study. The assumptions considered included a 95% confidence level (CL) (Zα/2 = 1.96), a power of 80% (Zβ = 0.84), a case-control ratio of 1:2, and a 10% non-response rate. The proportion of controls exposed, which was 38.2%, and a minimum detectable odds ratio of 1.9 were derived from a study conducted at Hawassa University Comprehensive Hospital among T2DM patients [17]. Based on these considerations, the final sample size was computed to include 138 cases and 275 controls, resulting in a total of 413 participants.

### Sampling techniques and procedure

A simple random sampling method was used to select two public hospitals from those located in the Sidama Region: Hawassa University Comprehensive Specialized Hospital (HUCSH) and Yirgalem Hospital Medical College (YHMC). The calculated sample sizes for cases and controls were proportionally allocated to the selected hospitals using the proportional allocation to sample size method. Specifically, 94 cases and 187 controls were selected from HUCSH, while 44 cases and 88 controls were selected from YHMC.

### Data collection tool and procedure

A questionnaire adopted from WHO STEPS manual for non-communicable disease surveillance was administered through face-to-face interview for data collection [18]. The tool includes sociodemographic items, behavioral risk factors like tobacco use, alcohol consumption, Khat chewing, dietary habits like fruits and vegetable intake and physical activity and clinical items like history of chronic illness, family history of DM and hypertension and lipid abnormalities. In addition, anthropometric measurements, blood pressure measurement and laboratory samples were used to collect the data.

### Blood pressure measurement

A calibrated Boso Medicus Uno instrument was used to take measurements while participants remained seated in a relaxed position, with their arm comfortably rested at heart level. Measurements were taken after a five-minute rest, with two readings recorded at three-minute intervals; a third reading was taken if the difference exceeded 5 mmHg. Hypertension was defined as systolic blood pressure ≥ 140 mmHg, diastolic blood pressure ≥ 90 mmHg [19, 20].

### Anthropometric measurement

T2DM patients’ weight was measured using a SECA digital scale (Seca GmbH & Co. KG, Germany), without shoes and with light clothing, to the nearest 100 g. The scale was placed on a flat surface. Height measurement was conducted using a measuring tape attached to a vertical wall, with a horizontal headboard touching the highest point of the head. Height was recorded in meters to the nearest 0.1 cm, while barefoot or in thin socks.

The body mass index (BMI) computation involved taking a T2DM patient’s weight in kilograms and dividing it by their height in meters multiplied by itself (kg/m²). Study participants were classified based on BMI: ≥30 kg/m² indicated obesity, 25–29.9 kg/m² signified overweight, while 18.5–24.9 kg/m² represented normal weight [21].

Waist circumference was measured at the midpoint between the lower rib margin and the iliac crest using a non-stretchable measuring tape, with participants standing upright and breathing normally. Two consecutive measurements were taken to the nearest 0.1 cm, and the average was recorded; a third measurement was performed if the first two differed by more than 1 cm. Waist circumference readings were assessed using the WHO-defined threshold values. According to these standards, a waist circumference above 94 cm for males or 80 cm for females is considered abnormal [22].

Hip circumference was measured using a flexible measuring tape positioned around the widest part of the hips. Participants were instructed to stand straight with their feet together while the tape was snugly applied but not compressing the skin. The measurement was recorded to the nearest centimeter at the end of a normal expiration to ensure accuracy. Based on these standards, a waist-hip ratio of ≥ 0.90 for males and ≥ 0.85 for females is considered abnormal [22].

### Blood sample collection and serum biochemical determination

About 5 milliliters of blood sample was collected following minimum of eight hour fasting or early in the morning before breakfast for fasting blood glucose (FBG), high density lipoprotein cholesterol, triglyceride and total cholesterol tests. Samples was collected with standardize serum separator tube by trained lab technician. The process of blood sample collection follows a sterile technique. Serum was obtained from collected blood sample by centrifugation at 3000 revolutions per minute for 10 min. The separated serum samples were analyzed for biochemical analysis using the A25TM BioSystem Random Access chemistry analyzer. During the series of biochemical analyses, two levels of quality control (PreciControl Clinical Chemistry Multi 1 and 2) were employed.

### Operational definition

#### Metabolic syndrome

This study used the IDF criteria to define MetS. This definition identified central obesity as an essential component of MetS and defined MetS as central obesity (based on race- and gender-specific waist circumference cutoffs with waist circumference ≥ 94 cm for male and ≥ 80 cm for female) plus any two of the following four factors: raised triglycerides (≥ 150 mg/dL (1.7 mmol/L) or specific treatment for this lipid abnormality), reduced high-density lipoprotein cholesterol (< 40 mg/dL (1.03 mmol/L) in male < 50 mg/dL (1.29 mmol/L) in female or specific treatment for this lipid abnormality), raised blood pressure (systolic blood pressure ≥ 130 mm Hg or diastolic blood pressure ≥ 85 mm Hg, or any patient on treatment of previously diagnosed hypertension), and/or raised fasting plasma glucose (≥ 100 mg/dl (5.6mmol/l) or with a previous history of diabetes) [6].

Glycemic control among patients with T2DM was defined using glycated hemoglobin (HbA1c) thresholds in accordance with the American Diabetes Association recommendations. An HbA1c value of less than 7% was considered indicative of good glycemic control, whereas an HbA1c value greater than 7% was categorized as poor glycemic control [23].

Physical activity among patients with T2DM was assessed using the Global Physical Activity Questionnaire developed by the WHO [24]. Total physical activity was calculated as Metabolic Equivalent of Task (MET) minutes per week by summing activity across the GPAQ domains. Based on established WHO guidelines, physical activity levels were categorized as low (0–600 MET-min/week), moderate (601–3000 MET-min/week), and high (≥ 3000 MET-min/week) [25].

### Data quality control

Qualified data collectors and supervisors received comprehensive two-day training on the Kobo Toolbox system and interview skills. The data collection tool was pre-tested on 26 (5%) type T2DM (8 cases and 16 controls) at *Leku General Hospital*, and necessary modifications were made. T2DM patients’ anthropometric measurements were taken using calibrated scales. Rigorous supervision included daily examinations and prompt error corrections. The laboratory equipment was checked and calibrated daily before new measurements were done. The use of the Kobo Toolbox system for data collection facilitated logical data entry and maintained data quality. Supervisors verified form completeness, ensuring data integrity throughout the process.

### Data analysis and interpretation

Data was collected using the Kobo Toolbox and exported into Statistical Package for the Social Sciences (SPSS) software version 26 for analysis. Measures of dispersion, central tendency, and frequency distributions were employed to characterize the data. The normality of distribution of continuous variables was assessed using the Kolmogorov-Smirnov and Shapiro-Wilk tests. Chi-square tests or independent t-tests were used to compare the socio-demographic and economic characteristics of the two groups, depending on the nature of the variables. BMI was calculated as weight in kilograms divided by the square of height in meters (kg/m²).

Bivariate and multivariable logistic regression analyses were conducted to identify predictors of MetS. Variables with a p-value < 0.25 in the bivariate model were included in the multivariable logistic regression model to mitigate the impact of confounding variables. The model’s fitness was assessed using the Hosmer and Lemeshow tests (*P* = 0.793), while multicollinearity was determined using the variance inflation factor (VIF ≤ 1.231). Statistical significance in the multivariable analysis was declared at a p-value < 0.05. Findings were presented AOR and 95% CI.

## Results

### Sociodemographic characteristics of study participants

A total of 400 T2DM patients (132 cases and 268 controls) participated in the study, with a response rate of 96.9%. The mean age (± SD) of the cases and controls was 56.9 (± 8.2) and 49.5 (± 8.1) years, respectively (*P* < 0.001). Slightly less than half (44.0%) of participants in the MetS group were aged over 61 years, compared to only 17.5% of subjects without MetS (*P* < 0.001). MetS was significantly more common among females (61.4%) than males (38.6%) (*P* < 0.001). MetS was more frequently observed among urban residents (77.3%) compared to those who resided in rural areas (22.7%) (*P* < 0.001). The two groups showed significant differences in terms of occupational status and monthly income level (*P* < 0.001) (Table 1).

**Table 1:** Sociodemographic characteristics of T2DM patients with or without MetS attending diabetic clinics of public hospitals in Sidama Region, Ethiopia

Variables	Cases (*n* = 132)		Controls (*n* = 268)		*P*-value
	Number	Percent (%)	Number	Percent (%)	
Age category in years					
≤ 45	18	13.6	101	37.7	< 0.001
46–60	56	42.4	120	44.8	
≥ 61	58	44.0	47	17.5	
Sex					
Male	51	38.6	193	72	< 0.001
Female	81	61.4	75	28	
Marital status					
Single	13	9.8	58	21.6	0.011
Married	85	64.4	165	61.6	
Divorced	19	14.4	22	8.2	
Widowed	15	11.4	23	8.6	
Place of residence					
Urban	102	77.3	127	47.4	< 0.001
Rural	30	22.7	141	52.6	
Occupation					
Housewife	25	18.9	52	19.4	< 0.001
Farmer	9	6.8	81	30.2	
Merchant	38	28.8	34	12.7	
Gov’t Employee	45	34.1	73	27.3	
Daily laborer	8	6.1	14	5.2	
Self employed	7	5.3	14	5.2	
Monthly income					
≤ 3500 Eth Birr	20	15.2	154	57.5	< 0.001
> 3500 Eth Birr	112	84.8	114	42.5	

### Behavioral characteristics of T2DM patients with and without MetS

T2DM patients with MetS smoked cigarettes more frequently than the controls (cases who ever smoked: 13.6% vs. controls who ever smoked: 5.2%, *P* = 0.040; cases with current smoking: 12.1% vs. controls with current smoking: 5.2%, *P* = 0.014). Alcohol consumption in the last 12 months was more common in T2DM patients with MetS (15.9%) compared with patients without MetS (9.3%) (*P* = 0.042). Similarly, alcohol use in the past 30 days was significantly more common in MetS group compared to controls (15.1% vs. 7.9%, *P* = 0.023). Lifetime khat chewing and khat chewing in the last 12 months did not show a significant difference between the groups (*P* > 0.05) (Table 2).

Based on WHO recommendations, dietary patterns indicated poor overall fruit and vegetable consumption among study participants. Concerning total weekly fruit and vegetable consumption among T2DM patients, mean intake was significantly lower in patients with MetS (8 ± 7 servings) compared with those without MetS (18 ± 11 servings; *P* < 0.001). Daily fruit and vegetable consumption (servings) did not show a significant difference between the groups (*P* = 0.061). T2DM patients with MetS reported spending significantly less time on moderate and high levels of physical activity compared to those without MetS. More than two-thirds (69.7%) of T2DM patients with MetS were categorized as having low physical activity, compared to 40.3% of those without MetS (*P* < 0.001) (Table 2).

**Table 2:** Behavioral characteristics of T2DM patients with or without MetS attending diabetic clinics of public hospitals in Sidama Region, Ethiopia

Variables	Cases (*n* = 132)		Controls (*n* = 268)		*P*-value
	Number	Percent (%)	Number	Percent (%)	
Ever smoked cigarette					
Yes	18	13.6	14	5.2	0.040
No	114	86.4	254	94.8	
Current smoker					
Yes	16	12.1	14	5.2	0.014
No	116	87.9	254	94.8	
Ever drank alcohol					
Yes	24	18.2	30	11.2	0.054
No	108	81.8	238	88.8	
Drank alcohol in the past 12 months					
Yes	21	15.9	25	9.3	0.042
No	111	84.1	243	90.7	
Drank alcohol in the past 30 days					
Yes	20	15.1	21	7.9	0.023
No	112	84.9	247	92.1	
Ever chewed Khat					
Yes	17	12.9	26	9.7	0.335
No	115	87.1	242	90.3	
Chewed Khat in the past 12 months					
Yes	15	11.4	24	9	0.445
No	117	88.6	244	91	
Daily fruit and vegetable serving					
< 5 Serving	132	100	261	97.4	0.061
≥ 5 Serving	0	0	7	2.6	
Weekly fruit & veg. serving (Mean ± SD)					
	8 ± 7		18 ± 11		< 0.001
Physical activity					
Low	92	69.7	108	40.3	< 0.001
Moderate	27	20.5	91	34.0	
High	13	9.8	69	25.7	

### Medical related characteristics of T2DM patients with and without MetS

About one-fifth of T2DM patients with MetS (22%) and 13.4% without MetS have been diagnosed with chronic diseases other than diabetes mellitus and hypertension (*P* = 0.030). One-fifth (20.5%) of T2DM patients with MetS and 10.1% without MetS received drug treatment for elevated cholesterol levels or dyslipidemia in the last 2 weeks (*P* = 0.004). Among T2DM patients with MetS, 46.2% reported a past history of raised blood pressure or hypertension compared to 29.9% in the controls (*P* = 0.001). Fifty-one T2DM patients with MetS (38.6%) reported that they have hypertension or raised blood pressure in the past 12 months compared with seventy-two (26.9%) without MetS (*P* = 0.016). The use of antihypertensive drugs in the last two weeks was significantly more common in the MetS group (37.1% vs. 22.4%, *P* = 0.001). Family history of hypertension was more common in T2DM patients with MetS (37.1%) than in those without MetS (16.4%) (*P* < 0.001) (Table 3).

About half (48.5%) of T2DM patients with MetS and 29.1% without MetS had a family history of DM (*P* < 0.001). The mean (± SD) duration with DM was 7.0 ± 1.4 years among patients with MetS and 5.0 ± 1.3 years without MetS (*P* < 0.001). More than half (59.1%) of T2DM patients with MetS and 43.7% without MetS had a duration of 5–9 years with DM (*P* < 0.001). The vast majority of T2DM patients (87.9% with MetS and 92.2% without MetS) were on DM medication in the past 2 weeks (*P* = 0.164). Nearly one-third (32.6%) of T2DM patients with MetS and one-fifth (20.5%) of those without MetS were currently using insulin as DM medication, which was significantly higher among MetS patients (*P* = 0.005) (Table 3).

**Table 3:** Medical characteristics of T2DM patients with or without Met attending diabetic clinics of public hospitals in Sidama Region, Ethiopia

Variables	Cases (*n* = 132)		Controls (*n* = 268)		*P*-Value
	Number	Percent (%)	Number	Percent (%)	
Chronic diseases other than THN & DM					
Yes	29	22	36	13.4	0.030
No	103	78	232	86.6	
Drug treatment for elevated cholesterol level or dyslipidemia in the last 2 weeks					
Yes	27	20.5	27	10.1	0.004
No	105	79.5	241	89.9	
Past history of HTN or raised BP					
Yes	61	46.2	80	29.9	0.001
No	71	53.8	188	70.1	
Last 12 months Hx of HTN/ raised BP					
Yes	51	38.6	72	26.9	0.016
No	81	61.4	196	73.1	
On medication for HTN in past 2 weeks					
Yes	49	37.1	60	22.4	0.001
No	83	62.9	208	77.6	
Family history of hypertension					
Yes	49	37.1	44	16.4	< 0.001
No	83	62.9	224	83.6	
On medication for DM within past 2 weeks					
Yes	116	87.9	247	92.2	0.164
No	16	12.1	21	7.8	
On insulin treatment					
Yes	43	32.6	55	20.5	0.005
No	89	67.4	213	79.5	
Mean duration with DM (± SD)	7.0 ± 1.4		5.0 ± 1.3		< 0.001
Family history of DM					
Yes	64	48.5	78	29.1	< 0.001
No	68	51.5	190	70.9	

*Abbreviations*: *BP* Blood Pressure, *DM* Diabetes Mellitus, *HTN* Hypertension, *SD* Standard Deviation

### Anthropometric characteristics of T2DM patients with or without MetS

The distribution of BMI among our study participants indicated that 83.3% of patients with MetS fell into the overweight/obesity category with a BMI of ≥ 25.00 kg/m², compared to only 49.3% of those without MetS (*P* = 0.021). The waist-to-hip ratio also showed a statistically significant difference between the groups, with 88.7% of cases classified as high risk, while only 32.1% of the control group were categorized in this group (*P* = 0.016). The vast majority (96.2%) of T2DM patients with MetS and 19.8% of those without MetS showed an increased risk of developing metabolic complications based on the waist-to-height ratio (*P* < 0.001). About two-thirds of the T2DM patients with MetS (63.6%) had raised blood pressure/hypertension compared to 10.8% of without MetS (*P* = 0.018) (Table 4).

**Table 4:** Anthropometric characteristics of T2DM patients with or without MetS attending diabetic clinics of public hospitals in Sidama Region, Ethiopia

Variables	Cases (*n* = 132)		Controls (*n* = 268)		*P*-Value
	Number	Percent (%)	Number	Percent (%)	
BMI (kg/m²)					
< 25	22	16.7	136	50.7	0.021
≥ 25	110	83.3	132	49.3	
Waist to hip ratio (WHR)					
Low risk	4	3.0	104	38.8	0.016
Moderate risk	11	8.3	78	29.1	
High risk	117	88.7	86	32.1	
Waist to height ratio (WHtR)					
Low risk	5	3.8	215	80.2	< 0.001
High risk	127	96.2	53	19.8	
Central obesity according to IDF					
Normal	0	0	254	94.8	< 0.001
Obese	132	100	14	5.2	
Blood pressure (mmHg)					
Normal	48	36.4	239	89.2	0.018
Raised	84	63.6	29	10.8	

*Abbreviations*: *BMI* Body Mass Index, *IDF* International Diabetic Federation

### Biochemical characteristics of T2DM patients with or without MetS

In terms of lipid profiles, more than half of T2DM patients with MetS (59.8%) had total cholesterol levels ≥ 200 mg/dL, compared to 10.8% of those without MetS (*P* < 0.001). A half of T2DM patients with MetS (50%) had elevated triglyceride levels (≥ 150 mg/dL), whereas only 3.7% of those without MetS had this condition (*P* < 0.001). Regarding HDL, slightly less than three-fourths of T2DM patients with MetS (72%) and 39.6% of those without MetS had reduced HDL, with a statistically significant difference (*P* < 0.001). The vast majority of T2DM patients with MetS (93.9%) and half of those without MetS (49.3%) had abnormal blood lipid levels (*P* < 0.001). About one-fifth (22.7%) of patients with MetS and more than half (58.2%) of those without MetS had good glycemic control status based on HbA1c (*P* < 0.001) (Table 5).

**Table 5:** Biochemical characteristics of T2DM patients with or without MetS attending diabetic clinics of public hospitals in Sidama Region, Ethiopia

Variables	Cases (*n* = 132)			Controls (*n* = 268)		*P*-Value
	Number	Percent (%)		Number	Percent (%)	
Total Cholesterol						
< 200 mg/dL	53	40.2	239		89.2	< 0.001
≥ 200 mg/dL	79	59.8	29		10.8	
Triglyceride						
< 150 mg/dL	66	50.0	258		96.3	< 0.001
≥ 150 mg/dL	66	50.0	10		3.7	
HDL						
Normal	37	28.0	162		60.4	< 0.001
Reduced	95	72.0	106		39.6	
Dyslipidemia						
Normal	8	6.1	136		50.7	< 0.001
Dyslipidemia	124	93.9	132		49.3	
Glycemic control (HbA1c)						
Good (< 7%)	30	22.7	156		58.2	< 0.001
Poor (≥ 7%)	102	77.3	112		41.8	

### Predictors of MetS among type T2DM

Among the variables considered for the bivariable logistic regression analyses, ten independent variables that showed a p-value of less than 0.25 were selected as candidate variables for the multivariable model. In the multivariable logistic regression analysis, age, sex, place of residence, monthly income, family history of hypertension, duration of diabetes mellitus treatment, and glycemic control status were significant predictors (*p* < 0.05) of MetS.

The ultimate model showed that T2DM patients aged 61 years or older were about six times more likely to have MetS compared to those aged 45 years or younger. Female T2DM patients had about three times higher odds of having MetS than males. Residing in urban areas increased 2.59 times the odds of having MetS compared to rural residents. Study participants earning more than 3,500 Ethiopian Birr per month had about four times higher odds of developing MetS than those earning less.

T2DM patients with a family history of hypertension were 2.79 times more likely to have MetS than those without such a history. Longer duration of diabetes was also a significant predictor: compared with patients with diabetes duration < 5 years, those with 5–9 years and ≥ 10 years’ duration had about threefold and fourfold higher odds of developing MetS, respectively. Glycemic control status was a strong predictor: T2DM patients with poor glycemic control were about four times more likely to have MetS than those with good glycemic control (Table 6).

**Table 6:** Predictors of MetS among T2DM patients with and without MetS attending diabetic clinics of public hospitals in Sidama Region, Ethiopia

Variables	Cases(%)	Controls (%)	COR (95% CI)	AOR (95% CI)	*P*-Value
Age category					
≤ 45 Years	18 (13.6)	101 (37.7)	1.00	1.00	
46–60 Years	56 (42.4)	120 (44.8)	2.61 (1.44–4.74)	1.99 (0.95–4.19)	0.068
≥ 61 Years	58 (44.0)	47 (17.5)	6.92 (3.68–13.02)	5.74 (2.56–12.88)	< 0.001
Sex					
Male	51 (38.6)	193 (72)	1.00	1.00	
Female	81 (61.4)	75 (28)	4.08 (2.63–6.34)	2.91 (1.61–5.26)	< 0.001
Place of residence					
Urban	102 (77.3)	127 (47.4)	3.77 (2.25–6.05)	2.59 (1.41–4.75)	0.002
Rural	30 (22.7)	141 (52.6)	1.00	1.00	
Monthly income					
≤ 3500 ETB	20 (15.2)	154 (57.5)	1.00	1.00	
> 3500 ETB	112 (84.8)	114 (42.5)	7.56 (4.43–12.90)	4.30 (2.23–8.28)	< 0.001
Current smoker					
Yes	16 (12.1)	14 (5.2)	2.50 (1.18–5.29)	2.91 (0.97–8.74)	0.057
No	116 (87.9)	254 (94.8)	1.00	1.00	
Diagnosed chronic Ds excluding HTN & DM					
Yes	29 (22)	36 (13.4)	1.81 (1.06–3.12)	1.07 (0.59–1.92)	0.833
No	103 (78)	232 (86.6)	1.00	1.00	
Family Hx of HTN					
Yes	49 (37.1)	44 (16.4)	3.00 (1.86–4.88)	2.79 (1.47–5.29)	0.002
No	83 (62.9)	224 (83.6)	1.00	1.00	
Family Hx of DM					
Yes	64 (48.5)	78 (29.1)	2.29 (1.49–3.53)	1.07 (0.59–1.92)	0.833
No	68 (51.5)	190 (70.9)	1.00	1.00	
Duration with DM					
< 5 years	22 (16.7)	120 (44.8)	1.00	1.00	
5–9 years	78 (59.1)	117 (43.6)	3.63 (2.12–6.22)	3.06 (1.57–5.99)	0.001
≥ 10 years	32 (24.2)	31 (11.6)	5.63 (2.87–11.01)	3.61 (1.54–8.48)	0.003
Glycemic control status					
Good	30 (22.7)	156 (58.2)	1.00	1.00	
Poor	102 (77.3)	112 (41.8)	4.74 (2.95–7.61)	3.93 (2.17–7.13)	< 0.001

*Abbreviations*: *AOR* Adjusted odds ratio, *COR* Crude odds ratio, *DM* Diabetes mellitus, *Ds* Disease, *ETB* Ethiopian Birr, *HTN* Hypertension, *Hx* History, *Tx* Treatment

## Discussion

A facility-based unmatched case-control study was conducted in the public hospitals of the Sidama Region to assess predictors of MetS among T2DM patients attending diabetic clinics. The study identified age, sex, place of residence, monthly income, family history of hypertension, duration of diabetes mellitus treatment, and glycemic control status as independent predictors of MetS among T2DM patients.

The present study found that the prevalence of MetS was higher among females (61.4%) than males (38.6%), with females having 2.92 times higher odds of developing MetS (95% CI: 1.61, 5.26). In line with our finding, previous studies conducted in Tigray, Ethiopia [26], Dessie, Ethiopia [27], and Northwest Ethiopia [28] reported 1.93, 2.43, and 3.98 times increased odds of developing MetS among female T2DM patients, respectively. The possible explanation could be due to the fact that, in many developing settings, including Ethiopia, women often engage in limited physical activity due to domestic responsibilities, contributing to a more sedentary lifestyle [29]. Physiological changes associated with pregnancy, including weight gain and gestational diabetes, may elevate the risk of MetS. Postpartum behaviors, such as consumption of high-fat diets and reduced physical activity, further increase the likelihood of abdominal obesity and difficulty in returning to pre-pregnancy weight [30]. Moreover, hormonal changes during premenopausal and menopausal stages may predispose women to central obesity and insulin resistance, thereby increasing the risk of MetS [31].

This study identified age as a significant predictor of MetS among patients with T2DM. Compared to individuals aged 45 years or younger, those aged 61 years and older had 5.74 times increased likelihood of having MetS (95% CI: 2.56, 12.88). Studies conducted in Addis Ababa [32], West Gojjam, Ethiopia [33], Northwest Ethiopia [28], Tigray, Ethiopia [26], Dessie, Ethiopia [27], and South Ethiopia [17] showed a consistent positive association (AOR > 1). These studies consistently reported that older participants had significantly higher odds of developing MetS compared to their younger counterparts.

This association may be attributed to age-related physiological and behavioral changes, including a decline in basal metabolic rate, reduced physical activity, and increased adoption of unhealthy lifestyles such as alcohol consumption [34]. Advancing age is also linked to greater accumulation of abdominal adipose tissue, which promotes insulin resistance [35]. Increased visceral fat releases free fatty acids into circulation, contributing to elevated triglycerides, higher low-density lipoprotein cholesterol levels, and overall increased risk of MetS [36].

Residing in urban areas was associated with 2.59-fold higher odds of developing MetS compared with living in rural areas in our study. This finding concurs with reports from studies conducted in Hawassa, Ethiopia [37], and Adama, Ethiopia [38], which similarly documented greater odds of developing MetS among urban dwellers. This association may be attributed to the obesogenic nature of urban environments, characterized by sedentary lifestyles (including physical inactivity and sedentary occupations), unhealthy dietary practices (particularly the consumption of energy-dense, nutrient-poor processed foods), and increased smoking and alcohol use. These factors are well-established contributors to the pathophysiological components of MetS, including insulin resistance, dyslipidemia, hypertension, and hyperglycemia [39]. Moreover, health system challenges in urban settings—such as fragmented care, limited preventive screening, and delayed diagnosis due to overburdened services—may further permit these metabolic abnormalities to progress unchecked [40].

The results of our study indicated that participants earning over 3,500 ETB per month had 4.3-fold increased odds of developing MetS compared to those with lower incomes (95% CI: 2.23, 8.28). This finding aligns with research conducted at Adama Hospital Medical College, which found that individuals with monthly incomes of 4,230 Ethiopian Birr and above had 5.87 times higher odds of MetS relative to the lowest income category [38]. Similarly, a study in Addis Ababa reported 3.31 times higher odds of developing MetS among T2DM patients who earned 4,000 or more Ethiopian Birr [32]. Higher income levels may be associated with increased intake of energy-dense, processed foods, more frequent consumption of meals outside the home, and lower levels of physical activity due to predominantly sedentary occupations and greater reliance on motorized transportation. Furthermore, higher income is often associated with urban residence, which may further promote sedentary behaviors and increase access to unhealthy dietary options [41, 42].

According to this study’s findings, patients with T2DM with a family history of hypertension were 2.79 times more likely to have MetS compared to those without such a history (95% CI: 1.47, 5.29). This is consistent with studies conducted in Adama, Ethiopia [38] and Tunisia [43], which found 2.65 and 2.21 times higher odds, respectively, of developing MetS among T2DM patients who had a family history of hypertension. This association may be explained by both genetic predisposition and shared lifestyle factors. Genetic factors can influence an individual’s susceptibility to components of MetS, particularly in those with T2DM and hypertension [44]. Another explanation is the shared lifestyle factors within families, such as similar dietary habits, which may contribute to the risk of developing MetS [45].

Likewise, the findings of our study revealed that a longer duration of diabetes is a significant predictor of MetS development. Participants with diabetes for 5 to 9 years and those with 10 years or more had 3.06 times (95% CI: 1.57, 5.99) and 3.61 times (95% CI: 1.54, 8.48) higher odds of developing MetS, respectively, compared to individuals with a diabetes duration of less than 5 years. This is consistent with studies conducted in Northwest Ethiopia [28], and Nepal l [46], which also reported a positive association between longer diabetes duration and 3.24 and 1.61 times higher odds of MetS among patients with T2DM, respectively.

The observed association between longer duration of diabetes and the development of MetS may be explained by the progressive nature of T2DM, whereby prolonged exposure to hyperglycemia and insulin resistance contributes to the accumulation of metabolic abnormalities characteristic of MetS. In addition, extended disease duration often coincides with cumulative behavioral and treatment-related challenges, such as declining adherence to lifestyle modifications and pharmacotherapy, which may further exacerbate cardiometabolic risk [4, 47].

Our study revealed that glycemic control status is an independent predictor of MetS among patients with T2DM. Participants with poor glycemic control were 3.93 times (95% CI: 2.17, 7.13) more likely to have MetS compared to those with good glycemic control. **A** consistent finding was reported from a study conducted in Northwest Ethiopia [28], which associated 2.53 times increased odds of MetS among T2DM patients with poor glycemic control. This increased risk arises because individuals with T2DM and poor glycemic control experience prolonged exposure to elevated blood glucose levels, leading to insulin resistance and increased fat accumulation [48]. Insulin resistance contributes to dyslipidemia and hypertension, which are key components of MetS, exacerbating the health risks associated with poorly managed diabetes [49]. Moreover, chronic hyperglycemia promotes inflammatory processes that may enhance metabolic dysregulation, perpetuating the cycle of poor glycemic control and increasing susceptibility to MetS [50].

This study has some limitations that should be considered when interpreting the findings. First, the hospital-based design, conducted in general public hospitals, may introduce selection and referral bias, as patients attending these facilities are more likely to have complicated, long-standing, or poorly controlled diabetes. This may limit the generalizability of the findings to the broader population of T2DM in the community, particularly those managed at primary care level or not engaged in regular follow-up. Second, the unmatched case–control design limits the ability to establish temporality between exposure and outcome, precluding definitive causal inference. Moreover, residual confounding from unmeasured variables cannot be entirely excluded despite multivariable adjustment.

## Conclusion

This study identified advanced age, female sex, urban residence, higher monthly income, family history of hypertension, longer duration of diabetes, and poor glycemic control status as independent predictors of MetS among patients with T2DM.

### Recommendations

Targeted screening strategies should be strengthened within diabetic clinics, with particular emphasis on high-risk groups such as older adults, women, and urban residents, to enable early detection of MetS and related complications. Routine integration of MetS screening into standard diabetes care—alongside continuous monitoring of cardiometabolic indicators—may improve timely diagnosis and management. Preventive interventions should incorporate structured health education and counseling that address modifiable behavioral risk factors, enhance treatment adherence, and promote lifestyle modifications, including healthy diet and physical activity. Integrating metabolic risk management into routine diabetes care services could support comprehensive and coordinated management of patients with T2DM. Future studies employing prospective study designs are recommended to establish causal relationships and evaluate the effectiveness of these interventions.

## Data Availability

All the data generated or analyzed during this study are available from the principal investigator or from the corresponding author upon reasonable request.

## Abbreviations

AOR: Adjusted Odds Ratio
BMI: Body Mass Index
CI: Confidence Interval
CL: Confidence Level
COR: Crude Odds Ratio
HUCSH: Hawassa University Comprehensive Specialized Hospital
IDF: International Diabetes Federation
MetS: Metabolic Syndrome
SD: Standard Deviation
T2DM: Type II Diabetic Patients
WHO: World Health Organization
YHMC: Yirgalem Hospital Medical College
